# Anatomical three‐dimensional model with peri‐implant defect for in vitro assessment of dental implant decontamination

**DOI:** 10.1002/cre2.841

**Published:** 2024-01-31

**Authors:** Sadia Nazir Khan, Odd Carsten Koldsland, Hanna Tiainen, Carl Hjortsjö

**Affiliations:** ^1^ Department of Prosthetics and Oral Function, Faculty of Dentistry University of Oslo Oslo Norway; ^2^ Department of Periodontology, Faculty of Dentistry University of Oslo Oslo Norway; ^3^ Department of Biomaterials, Faculty of Dentistry University of Oslo Oslo Norway

**Keywords:** CADCAM, dental implants, mechanical decontamination, peri‐implantitis

## Abstract

**Objectives:**

Access to the implant surface plays a significant role in effective mechanical biofilm removal in peri‐implantitis treatment. Mechanical decontamination may also alter the surface topography of the implant, potentially increasing susceptibility to bacterial recolonization. This in vitro study aimed to evaluate a newly developed, anatomically realistic, and adaptable three‐dimensional (3D)printed model with a peri‐implant bone defect to evaluate the accessibility and changes of dental implant surfaces after mechanical decontamination treatment.

**Material and Methods:**

A split model of an advanced peri‐implant bone defect was prepared using 3D printing. The function of the model was tested by mechanical decontamination of the exposed surface of dental implants (Standard Implant Straumann AG) coated with a thin layer of colored occlusion spray. Two different instruments for mechanical decontamination were used. Following decontamination, the implants were removed from the split model and photographed. Image analysis and fluorescence spectroscopy were used to quantify the remaining occlusion spray both in terms of area and total amount, while scanning electron microscopy and optical profilometry were used to analyze alteration in the implant surface morphology.

**Results:**

The 3D model allowed easy placement and removal of the dental implants without disturbing the implant surfaces. Qualitative and quantitative assessment of removal of the occlusion spray revealed differences in the mechanism of action and access to the implant surface between tested instruments. The model permitted surface topography analysis following the decontamination procedure.

**Conclusion:**

The developed 3D model allowed a realistic simulation of decontamination of implant surfaces with colored occlusion spray in an advanced peri‐implant defect. 3D printing allows easy adaptation of the model in terms of the shape and location of the defect. The model presents a valuable tool for in vitro investigation of the accessibility and changes of the implant surface after mechanical and chemical decontamination.

## 1 INTRODUCTION

Despite high survival rates, dental implants encounter biological complications, including peri‐implant bone loss. Peri‐implantitis affects 20%–35% of implant patients and 10%–20% of implants, varying with disease classification (Atieh et al., 2013; Kordbacheh Changi et al., 2019; Romandini et al., 2021). Effective decontamination of implant surfaces and complete biofilm removal is essential in treating peri‐implantitis and sustaining healthy peri‐implant tissues (Costa et al., 2018).

Several chemical, mechanical, and laser treatments have been suggested for disrupting bacterial plaque on implant surfaces (Verket et al., 2023). The most proposed devices for mechanical decontamination include titanium and plastic curettes, air abrasives, and ultrasound devices. These instruments can be used alone or combined and may also act as carriers for topical antimicrobial agents. However, the synergistic effect of mechanical nonsurgical or surgical treatments combined with adjunctive therapies has demonstrated limited efficacy (Ramanauskaite et al., 2021). Access to all implant surfaces is crucial for reducing the bacterial load, as residual biofilm and hard deposits on the implant surface may sustain inflammation in the peri‐implant soft and hard tissues.

The hard tissue morphology of the bone defect and other local factors impact the clinical outcome of decontamination treatment (Monje, Pons, et al., 2019; Schwarz et al., 2010). Chronic inflammation may cause anatomical changes resulting in soft tissue recession, bone configuration changes, and intraorally exposed implant surface. Since peri‐implant bone defects vary in configuration and severity (Schwarz et al., 2010), it is uncertain whether the tools for mechanical decontamination can access all areas of the implant surface for effective biofilm removal.

The decontamination capability of commercially available devices has been investigated in several in vitro and in vivo studies. In vitro decontamination studies have been performed on titanium objects such as discs, coins, sheets, healing abutments, and various other objects simulating the dental implant surface (John et al., 2014; Louropoulou et al., 2014; Pereira da Silva et al., 2005; Sánchez et al., 2011). Studies evaluating decontamination effectiveness on dental implants have been conducted on implants alone or implants inserted in blocks of different materials (Bermejo et al., 2019; Cha et al., 2019; Keim et al., 2019; Nemer Vieira et al., 2012). In recent publications, three‐dimensional (3D) blocks holding dental implants have been designed with different bone defect configurations to evaluate the decontamination effectiveness in the presence of varied bone defect anatomies and teeth (Keim et al., 2019; Korello et al., 2023; Luengo et al., 2022; Ronay et al., 2017; Steiger‐Ronay et al., 2017). While these models allow for decontamination assessment in the presence of various defect angulations and configurations, they may not fully capture the complexity of real‐life clinical situations, where access to one implant surface may be more challenging than others due to differences in defect morphology on site level and the presence of adjacent teeth. Anatomically realistic in vitro 3D models provide a valuable tool for evaluating decontamination effectiveness at the site level. Furthermore, these models may serve as educational tools, providing students and professionals with an enhanced understanding of decontamination and its associated challenges.

Computer‐aided design and manufacturing (CADCAM) combined with 3D printing enables the fabrication of accurate models of complex objects (van Noort, 2012). CADCAM facilitates the modification of peri‐implant defects, tooth anatomy, implant angulation, and implant‐retained supraconstructions, simulating various clinical scenarios. Models with anatomical design, nonsymmetrical peri‐implant defects, and access to remove the implant without disturbing the implant surface have not been manufactured or evaluated in the published literature.

This study aimed to assess the feasibility of an anatomically realistic in vitro 3D‐printed model with an advanced peri‐implant defect to evaluate access and mechanical damage to implant surfaces caused by different tools for mechanical decontamination.

## 2 MATERIALS AND METHODS

### 2.1 In vitro 3D model design and manufacturing

**Figure 1:**
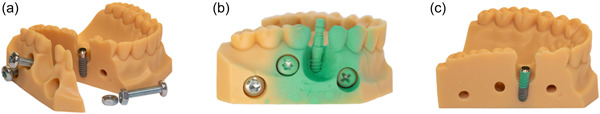
Anatomically realistic, three‐dimensional, printed resin model with an advanced peri‐implant defect. The model was designed with a split design for easy access to implant removal. (a) The dental implant was inserted into the split model and the parts were fixed with screws. (b) The exposed implant was sprayed with the colored occlusion spray after the model had been assembled. (c) The front panel of the model was unmounted for easy removal of the implant. The colored spray was not spread beyond the exposed area.

**Figure 2:**
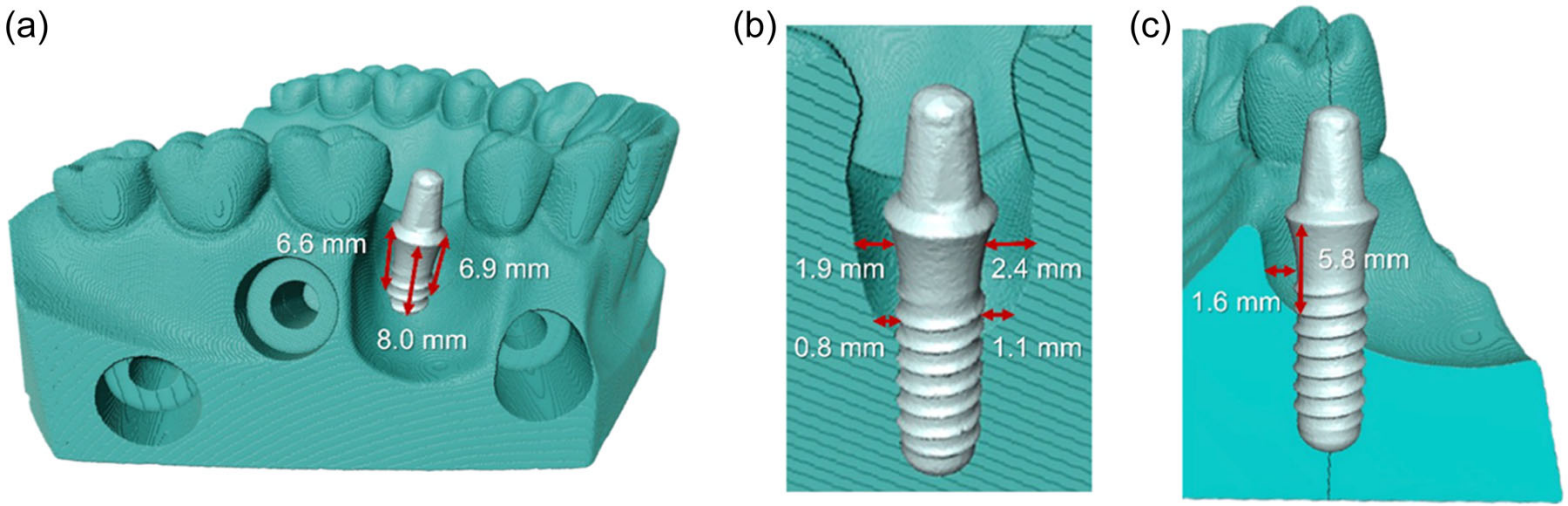
Peri‐implant defect dimensions measured with the computer‐aided design software (mm). (a) Vertical height at the mesial, buccal, and distal implant surface. (b) Horizontal defect dimensions at the second thread and the implant neck. (c) Vertical and horizontal defect dimensions measured at the palatal implant surface.

A phantom dental model (N222; Colombia Dentoform Corporation) was scanned using a workbench scanner (S600 ARTI, Software Zirkonzahn.Scan, Version 5051; Zirkonzahn®). One premolar was removed digitally with a computer‐aided design (CAD) program (Zirkonzahn.Archive, Version 7058; Zirkonzahn®). A 3b bone defect was designed in the edentulous premolar area as described by Monje, Pons et al. (2019). A titanium dental implant (Standard Implant, Ø 4.1 mm RN, SLA® 12 mm; Straumann AG) was scanned. The phantom dental model scan and the dental implant scan were superimposed in a planned position (Zirkonzahn.Modellier, Version 6173_6958_x64, Zirkonzahn®). The phantom model was split into two at the center of the occlusal surface of the teeth (Meshmixer™, Version 3.5.474; Autodesk®). A volume according to the scanned dental implant was stamped out digitally (Zirkonzahn.Modifier, version v.21.3_6.25448; Zirkonzahn®). STL files were forwarded to a slicer (CHITUBOX V1.8.1) and to a 3D printer (Phrozen Sonic XL 4K; Phrozen Tech Co., Ltd). The 3D models were printed in a resin material (Fotodent® model, 385/405 nm, Ref. D35400, Dreve [*n* = 12]). The printed models were rinsed with 95% ethanol (Antibac®) and a liquid for solvent cleanser (IMPRIMO® Cleaning Liquid; SCHEUGROUP) in an ultrasonic bath for 40 min (Model no. UCI‐230, SERIAL NO 931236817; Colténe/Whaledent Inc.). After cleansing, the models were light cured (Photopol. 230 V‐50/60 Hz 300 W; Dentalfarm) with light‐emitting diode (LED)/UV cure replacement bulbs (LED 9 W 405 nm; FEPshop BV). The cured models were assembled (Figure 1a) and scanned using the same workbench scanner as previously introduced (*n* = 5). Scans were superimposed to assess the similarity of the printed 3D models using the same CAD program as used for designing the 3D model. The dimensions of the peri‐implant defect surrounding the inserted implant were digitally measured using the same software (Figure 2).

Sterile titanium dental implants (Standard Implant, Ø 4.1 mm RN, SLA® 12 mm; Straumann AG) (*n* = 36). The implants were carefully mounted to the split models, and the parts were put together with nuts and bolts (DIN 934 M4 G 340004&M4X20; Arvid Nilsson). The exposed implant surface was coated with a thin even layer of colored occlusion spray 1 h before decontamination treatment (Okklufine Premium; FINO GmbH) (Figure 1b,c). The film thickness was assessed by using optical profilometry (S neox, Sensofar). For this purpose, a small area on the machined implant collar was masked using polyvinyl siloxane impression material (Provil® novo; Heraeus) during the application of the occlusion spray layer on the implant surface (*n* = 3). The mask was carefully removed, and three nonoverlapping areas (0.87 × 0.65 mm^2^) of the boundary region between the sprayed and unsprayed implant surface were imaged on each sample in confocal mode using red light and EPI ×20 objective. The average step height between the spray film and the masked area was then measured from each recorded image (*n* = 9) (Sensomap Standard 7.4; Sensofar).

### 2.2 Decontamination strategy

To test the ability of the model to reveal differences in decontamination outcomes following various decontamination protocols, the exposed implant surface was mechanically decontaminated using decontamination methods with different modes of action operated by one experienced clinician using the following methods with implants receiving no decontamination treatment serving as controls (*n* = 6):

1. Oscillating chitosan brush (OCB; Bioclean®; Labrida).
2. Polyetheretherketone tip for an ultrasonic unit (US‐PEEK; PI Instrument®; E.M.S. Electro Medical Systems) with water irrigation.
3. Irrigation with water spray using a three‐way dental syringe (water).
  To assess the capacity of the model to show differences between treatment outcomes of additional chemical debridement treatments in combination with OCB, four additional groups were tested (*n* = 3):
4. OCB with water irrigation (OCB + water);
5. OCB with chlorhexidine gel (CG; Corsodyl®; 1% Dental Gel) (OCB + CG);
6. OCB with blank gel (BG; 5% (wt/vol) methylcellulose; Sigma‐Aldrich) dissolved in deionized water (OCB + BG).
7. CG (Corsodyl®; 1% Dental Gel).

All OCBs were soaked in 0.9% sodium chloride solution for 2 min before being attached to an oscillating handpiece (NSK) at 1000 revolutions per minute.

Decontamination was performed on the exposed part of the implant for 2 min for all treatment groups. Following decontamination, the 3D models were photographed before the implants were carefully dismounted from the open models without disturbing the implant surface. Remaining moisture and loose particles were removed from the implant surface using clean, pressured air before photographing the samples. The implants were numbered groupwise before decontamination assessment, and the operator performing the implant characterization was therefore blinded for treatment allocation.

### 2.3 Decontamination assessments

#### 2.3.1 Photographing image analysis

The implants were fixed in a custom‐made holder by a transfer piece and photographed from the buccal, distal, palatal, and mesial sites with a single‐reflex camera (Nikon D3200 equipped with a Nikon 105 mm f/2.8D AF Micro Nikkor macro lens). For this purpose, the transfer piece was marked on its buccal side with a marker pen before removing the implants from the model. Imaging was performed at a fixed angle perpendicular to the height axis of the implant using fixed imaging settings and lighting conditions. Images were calibrated for white balance and contrast (Adobe Lightroom Classic) and binarized based on color histograms (ImageJ®; Figure S1). Implant diameter was used to calibrate the scale of the images. Based on the recorded images, the area still covered with the occlusion spray was determined on site level using ImageJ (Schneider et al., 2012). To compensate for potential minor alterations in implant orientation and imaging angle between experiments and test groups, all quantitative results of the image analysis are presented as a ratio between the area covered with green occlusion spray and the total projected implant area for each implant in percentage. The analyzer was blinded to decontamination allocation. Image analysis was repeated three times for each group.

#### 2.3.2 Fluorescence spectroscopy

To assess the total amount of occlusion spray remaining on the implant surface, the implants were then unmounted from the holder and placed in 1.7 mL microcentrifuge tubes after carefully removing the transfer piece from the implants. The remaining occlusion spray film was removed by bath sonicating the implants in 1 mL isopropanol for 20 min at 60°C. To reduce solvent evaporation, the closed microtubes were sealed with Parafilm® M laboratory film (Bemis Company) for the duration of the sonication. The solvent containing the dispersed occlusion spray particles was collected and used to estimate the amount of remaining occlusion spray film using a fluorometer (Qubit 4; Invitrogen). Fluorescence values were read using blue LED excitation at 470 nm and recording emission at far red (665–720 nm) wavelengths and converted to wt/vol concentration via a standard curve recorded for serial dilutions of known concentration of the used fluorescent occlusion spray particles dispersed in the same solvent (Figure S2).

#### 2.3.3 Evaluation of surface topography

Following removal of the occlusion spray film from the implant surface, the implants were dried using clean pressured air and imaged using a tabletop scanning electron microscope (SEM; TM‐3030; Hitachi) and optical profilometer (S neox; Sensofar) to assess the extent of potential mechanical damage caused on the machined and SLA implant surface (*n* = 3). For SEM imaging, the samples were fixed on an aluminum holder using conductive carbon and adhesive copper tape and imaged by detecting backscattered electrons generated at 15 kV acceleration voltage. The surface topography of the decontaminated implants was further visualized and quantified using optical profilometry. An area of 0.87 × 0.65 mm^2^ at three randomly chosen nonoverlapping positions on the buccal surface of both the machined implant collar and the sandblasted and acid‐etched implant screw was imaged in confocal mode using EPI ×20 objective (*n* = 3). Image processing (form removal and Gaussian filter: nesting index 50 µm) and quantification of surface parameters were performed using SensoMap Standard 7.4 (Sensofar).

### 2.4 Statistical analysis

The implant area covered with residual occlusion spray versus the total implant surface was described using percentage. All image measurements were performed three times, and the means and standard deviations were calculated. The means and SD of the fluorescence measurements for each decontamination group and the control implants were calculated. For the optical profilometry data, the means and SD for the machined implant collar and the sandblasted acid‐etched implant screw were calculated. Differences between the decontamination groups and implant sites were tested using one‐way analysis of variance. Statistical significance was considered at *p* < .05. Statistical analyses were performed using SPSS (IBM Statistics, Version 28.0.1.1.14; IBM).

## 3 RESULTS

### 3.1 Peri‐implant defect model

**Figure 3:**
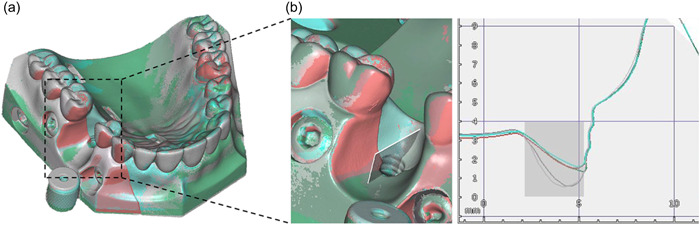
Evaluation of printed three‐dimensional (3D) model similarity using computer‐aided design software. (a) Five printed models were scanned and assigned five distinct color codes before they were superimposed. The superimposed models exhibited minimal variation in model parts or defect configurations. (b) Dimensional variations within digitally cut cross‐sections of the superimposed 3D scans of the printed model were limited to a few micrometers and are considered insignificant for the function of the model. These minimal variations are caused by shrinkage during final curing of the model and are smaller than the resolution of the 3D printing used to produce the models. Area highlighted in gray corresponds to the volume occupied by the implant and cannot therefore be scanned accurately due to restricted access to light within this area of the model during scanning.

The printed 3D models showed minimal variation in model parts and the defect area (Figure 3). All models showed the exact precise fit between the components. Implants were inserted identically, exposing the same number of threads at the respective surfaces. Buccally, the defect led to the exposure of four threads (8.0 mm). Distally and mesially, two threads (6.6 and 6.9 mm, respectively) and palatally one thread (5.8 mm) were exposed as illustrated in Figure 2, which also illustrates the horizontal dimensions of the peri‐implant defect. The amount of occlusion spray on the unexposed implant surface was minimal (Figure 1c). The film thickness of the occlusion spray layer on the implant surface was 9.6 ± 1.4 µm, as measured by optical profilometry and illustrated with SEM images in Figure S3. The occlusion spray was composed of solid inorganic particles embedded in a polymeric matrix and morphologically resembled a multispecies biofilm grown on titanium implant surface (Figure S3A–D).

### 3.2 Visual inspection and image analysis of the implant surface decontaminated mechanically

**Figure 4:**
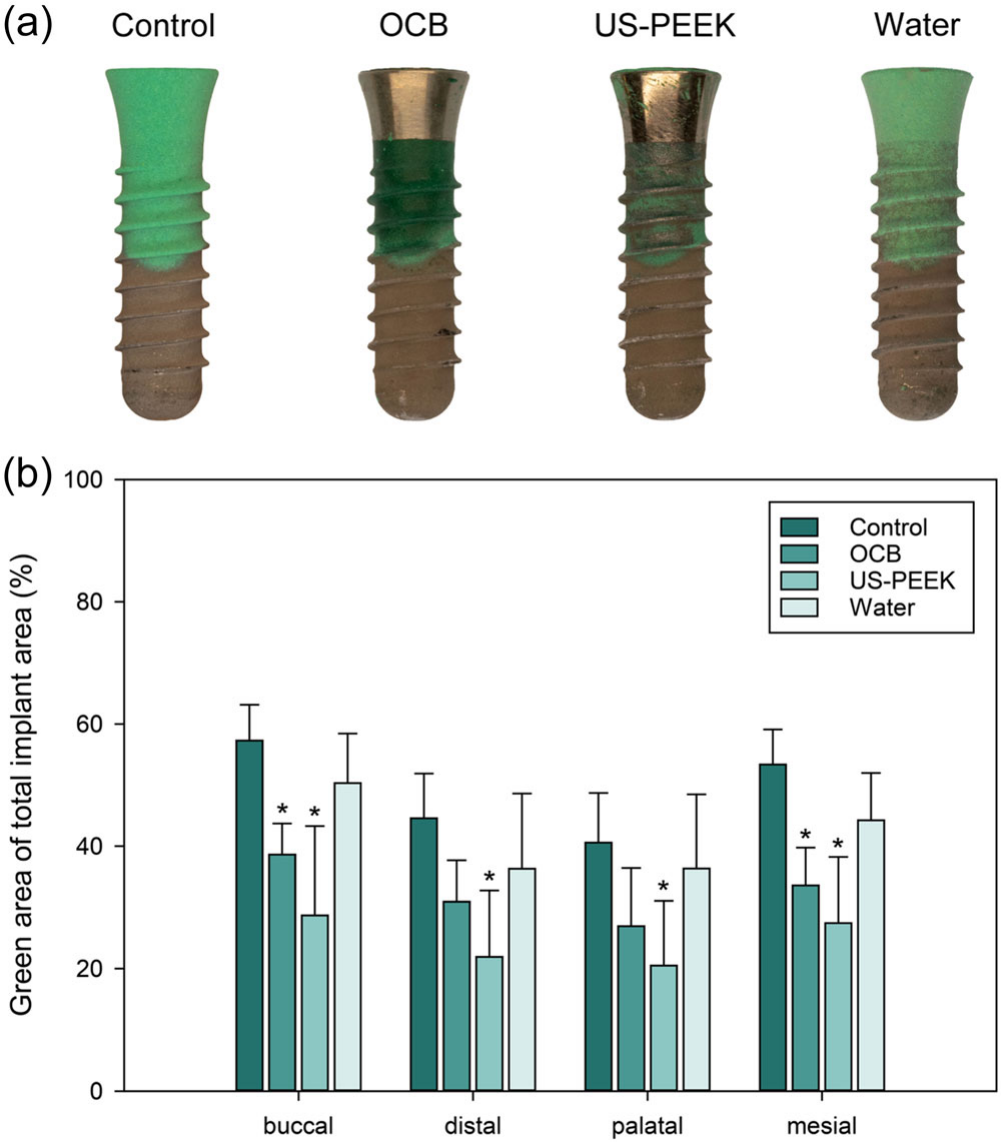
Image analysis of the area covered with residual occlusion spray versus total implant area. (a) The extent of occlusion spray coverage on the buccal surface of the control implant and changes in the occlusion spray coverage after mechanical decontamination treatment. (b) The ratio of area covered with remnant occlusion spray versus total implant area was calculated for control implants and implants treated with mechanical decontamination (*n* = 6, mean ± SD, *p < .05 compared to control at respective implant site). OCB, oscillating chitosan brush; US‐PEEK, ultrasonic polyetheretherketone tip.

Visual inspection of the implant surfaces in the control group demonstrated completely intact occlusion spray with a pattern analogous to the defect morphology (Figure 4a and Figure S4). A similar intact layer of green occlusion spray was observed on implant areas not reached by the decontamination instruments in the OCB and US‐PEEK groups, particularly in areas adjacent to the defect margins and the valley areas immediately below the threads in US‐PEEK samples.

Implants decontaminated with the OCB showed efficient removal of the green occlusion spray from the machined implant collar. However, the occlusion spray appeared visibly darker green for the threaded implant area compared to the control implants, indicating that the OCB accessed the entire surface but was not able to decontaminate the surface fully. In contrast, only partial removal of the colored occlusion spray with visible areas of intact spray layer at all four implant sites for both the machined collar and the threaded implant area was observed for the US‐PEEK group (Figure S4). Although the US‐PEEK instrument did not reach the entire surface, a significantly larger amount of green occlusion spray was removed from the threaded area compared to the OCB group (Figure 4b).

No decontamination method resulted in complete removal of the occlusion spray, as shown in Figure 4b. At the buccal and mesial implant surfaces, the removal of the occlusion spray showed statistical significance in the OCB and US‐PEEK groups when compared to the control group (*p* < .05). Notably, the occlusion spray removal was significantly higher in the US‐PEEK group compared to the control group at all four sites (*p* < .05), whereas there was no statistically significant difference between the water and control group at any implant site.

Overall, the implants in the water group demonstrated the least removal of the occlusion spray, with results resembling the control group. The green occlusion spray color in the water spray group was slightly lighter at both the implant neck and the threaded implant area compared to the control implants. Nevertheless, no implant areas were displayed without the occlusion spray in the water group, indicating that the occlusion spray was affected by the water but not removed.

### 3.3 Visual inspection and image analysis of combined chemical and mechanical decontamination

**Figure 5:**
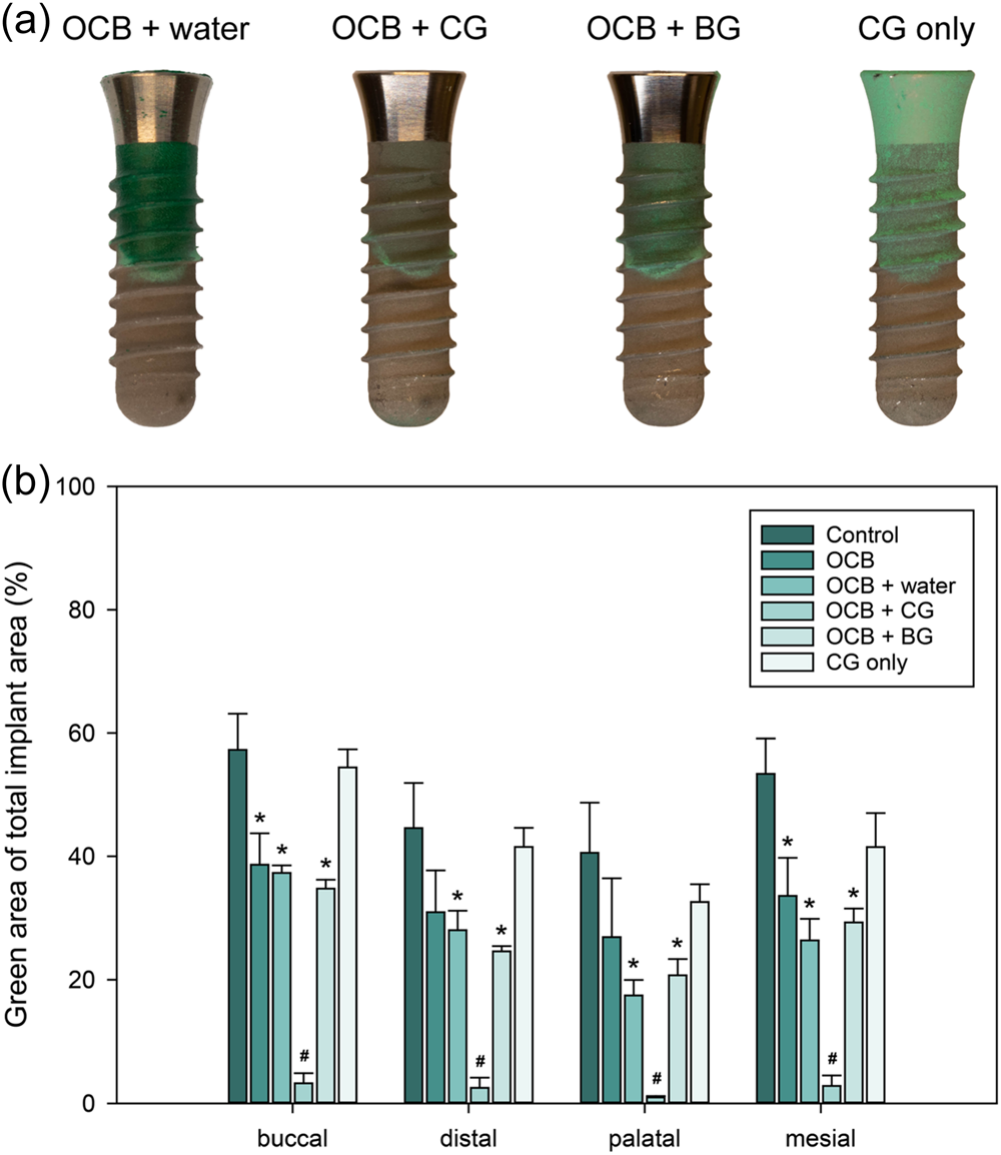
Qualitative and quantitative analysis of residual spray on implant surfaces. (a) Changes in the colored occlusion spray on the buccal implant surface after chemical or combined mechanical and chemical decontamination treatment. (b) Area with remnant occlusion spray versus total implant area for the control implant and implants decontaminated with chemical or combined chemical and mechanical decontamination (*n* = 3, mean ± SD, *p < .05 compared to control at respective implant site, ^#^ *p* < .05 compared to all other groups at respective implant site). BG, blank gel; CG, chlorhexidine gel; OCB, oscillating chitosan brush.

Equivalent to the water group, the occlusion spray was minimally affected by the CG, as shown in Figure 4 and Figure S5. Furthermore, OCB combined with water irrigation showed results similar to OCB without water irrigation, with a clean implant neck and a darker green occlusion spray layer at the threaded implant area. OCB combined with CG was the group that differed from all other decontamination groups as minimal to no occlusion spray remained at the machined implant neck and the threaded area after decontamination (Figure 5a and Figure S5). A clean implant neck and darker green occlusion spray on the threaded part were displayed when OCB was combined with BG. However, the darker green occlusion spray layer seemed thinner, and more of the implant surface was shown through the occlusion spray compared to the OCB group. The image analysis showed no statistically significant difference in the removal of the occlusion spray between the OCB combined with water or BG compared to OCB without irrigation at any of the four implant sites. For OCB combined with CG, the removal of occlusion spray was significantly higher compared to all other OCB groups as well as the CG and the control group for all implant sites (*p* < .05; Figure 5b).

### 3.4 Assessment of residual occlusion spray

**Figure 6:**
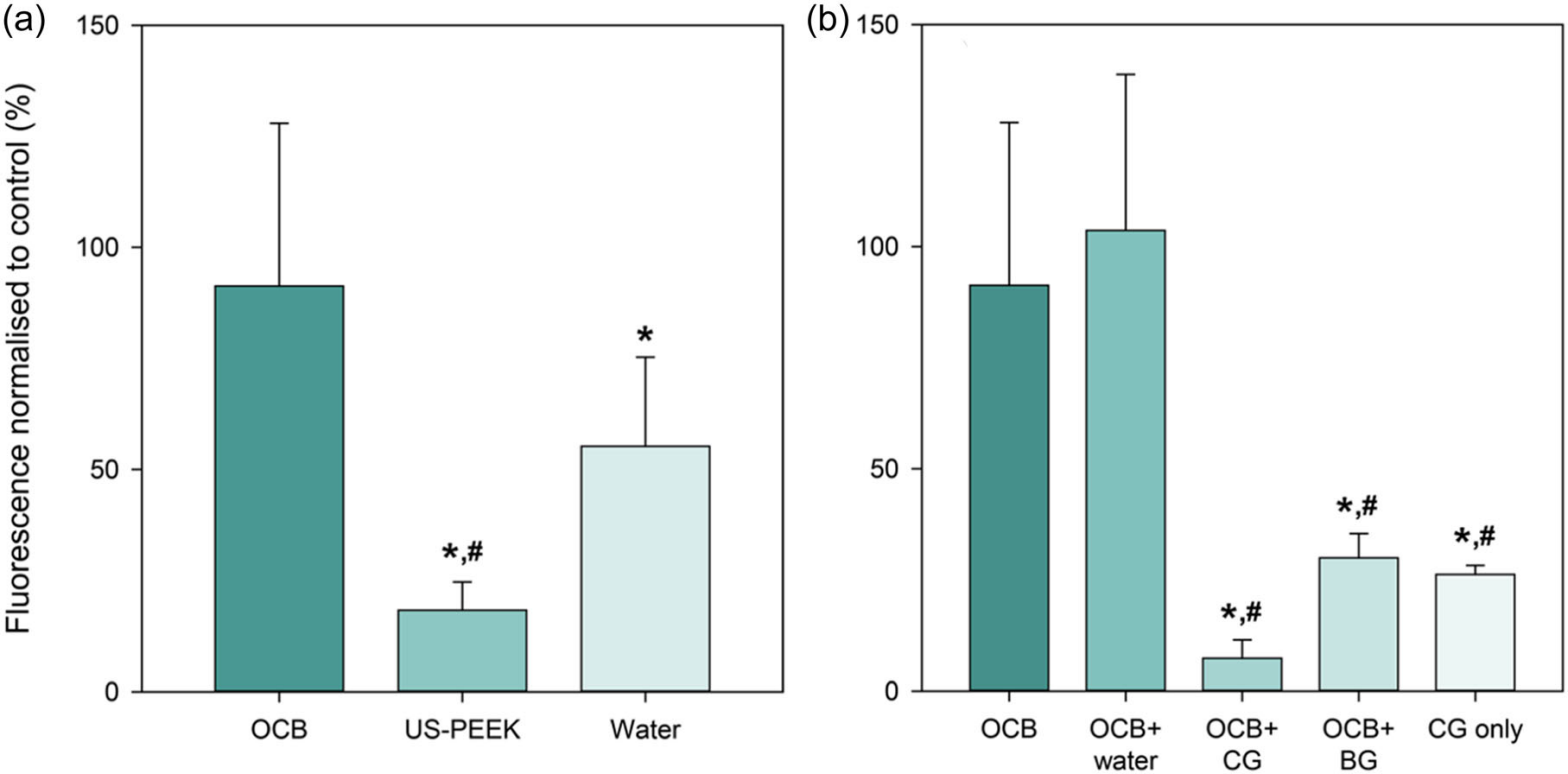
The mean fluorescence intensity of remnant occlusion spray indicating the total amount of unremoved colored occlusion spray on the decontaminated implant surface normalized to control (mean ± SD, (a): *n* = 6, (b) *n* = 3, *p < .05 compared to control, ^#^ *p* < .05 compared to OCB). BG, blank gel; CG, chlorhexidine gel; OCB, oscillating chitosan brush; US‐PEEK, ultrasonic polyetheretherketone tip.

Residual occlusion spray was removed from the implant surface with sonication in isopropanol. Fluorescence spectroscopy results showing the amount of residual occlusion spray on the implant surface after decontamination are displayed in Figure 6.

Implants decontaminated with OCB showed higher amount of residual occlusion spray than the water spray, control, and US‐PEEK group. The high fluorescence values were also demonstrated when OCB was combined with water due to difficulty of dispersing condensed residual occlusion spray detached from the implant surface. A decrease in the values was shown when OCB was combined with the BG. The lowest values were obtained for OCB combined with CG, corroborating the results from the image analysis.

### 3.5 Surface topography

**Figure 7:**
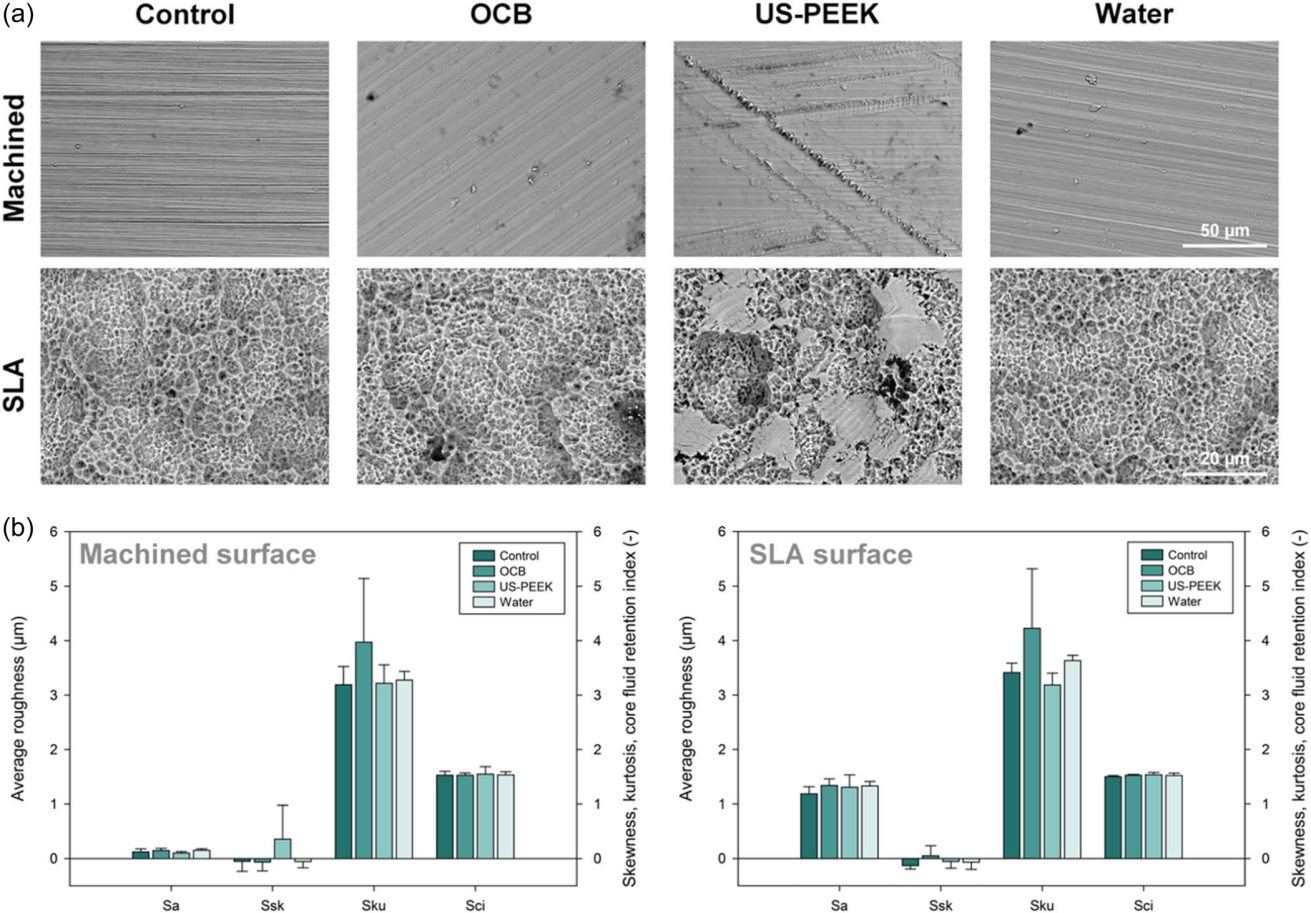
(a) Micromorphologic surface changes analyzed on scanning electron microscopy images (×2000 magnification) of an untreated control implant and implants after decontamination. Visible changes of surface topography were only observed in the US‐PEEK group. (b) Average surface texture parameters of the decontaminated machined implant neck and the SLA surface showed no remarkable changes in the overall surface topography. (*n* = 3, mean ± SD). OCB, oscillating chitosan brush; US‐PEEK, ultrasonic polyetheretherketone tip.

Figure 7a presents the SEM images for the control and the decontaminated machined and SLA surfaces. The images showed notable changes in the machined and threaded implant area in the US‐PEEK group, displayed as scratching and disruptions. The scratches were irregular in size and shape and distributed throughout the machined implant neck. For the threaded implant area, pronounced flattening of the microscale peaks on the sandblasted surface was observed, especially on the apex of the implant threads. OCB and water spray groups revealed no visible alterations in the surface texture of the machined implant neck or the SLA‐treated implant body.

The results obtained from the optical profilometry are displayed in Figure 7b. No statistically significant difference in the average surface roughness (Sa), surface skewness (Ssk), kurtosis (Sku), or core fluid retention (Sci) was observed between the groups for the machined or the SLA surface.

## 4 DISCUSSION

This study aimed to evaluate a CADCAM‐produced anatomical 3D model to investigate the efficacy of mechanical and chemical decontamination of dental implants. CADCAM production was selected because it allows easy modification of peri‐implant defect configuration and dimensions. Furthermore, the method facilitates the production of models with various nonsymmetrical defects, potentially within the same model. It also allows the design of bone defects based on data from cone beam computed tomography or intraoral scans obtained during surgical intervention. The precision and reproducibility of the models are assured by machine fabrication. The model can be designed for integration in a phantom head for educational purposes and hands‐on clinical training.

The effectiveness of tools for mechanical decontamination of dental implants has previously been investigated using 3Dprinted models with different standardized defect configurations as carriers for dental implants (Korello et al., 2023; Luengo et al., 2022; Matsubara et al., 2020). Contrary to the present study, the models were not designed anatomically with a split design. While evaluating decontamination using nonanatomical models with symmetrical defects is valuable, incorporating nonsymmetrical defects with realistic contours offers a more clinically relevant assessment of decontamination methods in vitro.

The bone defect shape is another factor influencing access to contaminated implant surfaces. Understanding the application of treatment methods in varying defect configurations may provide relevant knowledge for managing peri‐implant diseases (Keim et al., 2019; Luengo et al., 2022). Moreover, the accessibility of implant surfaces may differ at the site level, particularly in nonsymmetrical defects and the presence of teeth. The use of a model with a split design facilitates the evaluation of the implant on specific sites.

In addition to addressing bone defect morphology, replicating biofilms poses additional challenges, as simulations do not fully replicate the clinical situation and behave differently compared to natural biofilm. A common approach in in vitro studies to evaluate the efficacy of decontamination methods is using ink staining as a biofilm mimic (Luengo et al., 2022; Matsubara et al., 2020; Mensi et al., 2020; Ronay et al., 2017; Sahrmann et al., 2015). The simulated biofilms do not adhere to the implant surface in the same manner as natural biofilms. Additionally, using sprays and inks introduces a treatment bias as they are visible to the operator. However, this bias could be circumvented using colored filters that absorb light at the specific wavelengths of the ink or spray.

In the present study, *colored* occlusion spray, simulating biofilm, was applied to implant surfaces. In the control group, implants demonstrated consistent spray application to the exposed implant surface. The implants were removed, preserving intact occlusion spray and indicating no spray application on parts embedded in the model, as demonstrated in Figure 1. The model had a split design to facilitate the convenient insertion and removal of dental implants without disturbing the biofilm or coatings. This feature enables the reuse of the model components in multiple experiments, particularly with autoclavable resin.

Colored occlusion spray, containing microscale particles embedded in a polymeric matrix, was used to mimic biofilm (Figure S3). In composition, the occlusion spray is assembled as biofilm and may provide a better simulation of biofilm than ink (Figure S3,E,F). The use of colored occlusion spray to mimic biofilm was previously reported by Tuna et al. (2019) in an in vitro study with the aim of studying the removal of simulated biofilm on implant‐retained restorations.

Visual inspection of implants after decontamination showed various distinct differences in the residual occlusion spray layer for each tested decontamination method, demonstrating the capacity of this model to reveal differences in not only the access to the implant surface but also the mechanism of action and the efficacy of the different tested decontamination strategies in removing the occlusion spray from the surface. Implant areas that mechanical devices could not reach displayed an intact green shade similar to the spray layer on the control implants. In contrast, areas with complete decontamination had no residual occlusion spray. A dark, green, glossy surface of the residual occlusion spray layer was found on the SLA surface, regardless of whether or not water irrigation was used in the OCB groups (Figures 4 and 5). In the absence of any residual occlusion spray on the machined implant collar, this demonstrated that OCB reached the entire implant surface but was incapable of removing the spray in the micro‐rough surface of the implant body. Furthermore, the reduced thickness of the residual spray layer (Figure 6b) when combined with a gel (CHX or BG) was visually observed as a pale shade of green (Figures 5 and Figure S5), indicating that the gel was capable of assissting the mechanical decontamination process using this device. These results illustrates that the presented 3D model, combined with occlusion spray as biofilm mimic, allows to differentiate between restricted accessibility and limited decontamination capacity.

In vitro decontamination studies using ink as biofilm simulation report quantitative comparisons between the methods (Luengo et al., 2022; Matsubara et al., 2020). In the present study, residual green occlusion spray assessment following decontamination involved visual inspection, image analysis, and fluorescence spectroscopy. Furthermore, the present study demonstrates the implementation of fluorescence spectroscopy to analyze the total amount of occlusion spray remaining on implant surfaces, extending beyond assessments of the residual spray area analyzed through image analysis. Fluorescence evaluation distinctly differentiated the instruments' efficiency in removing or compressing the occlusion spray more tightly, as observed in the OCB group. Following the OCB decontamination process, the colored occlusion spray exhibited densely packed and adhered to the SLA surface, posing challenges during the sonication removal. The sonication procedure did not effectively break up the larger, condensed occlusion spray particles. Consequently, this caused an overestimation of the spray content, which was observed as elevated fluorescence levels in the OCB groups.

In the present study, two machine‐driven decontamination tools with distinct mechanisms of action were selected to assess the novel, anatomically realistic, 3D‐printed model. The defect configuration was designed to differ in depth and width across implant surfaces to evaluate the instruments' reach. In line with comparative studies utilizing ink (Luengo et al., 2022; Matsubara et al., 2020), the colored occlusion spray was not completely removed in any decontamination group. However, the decontamination groups demonstrated significantly better spray removal at the buccal and mesial implant sites than the control group (*p* < .05). For the decontamination methods, the US‐PEEK group performed significantly better in occlusion spray removal compared to the control groups at all four sites (*p* < .05), demonstrating the functionality of the model.

In contrast, decontamination with OCB exhibited greater efficiency in removing occlusion spray from the machined implant collar compared to the rough SLA implant surface. The residual occlusion spray for OCB remained prominent on all sites within the SLA area, however, with a changed green shade. The change in the green color indicated that the OCB reached all implant areas: threads, valleys, and flanks, but was unable to remove the green occlusion spray from the roughened SLA surface of the implant. This observation was further supported by the high fluorescence values, indicating aggregated spray material. These results show that the developed 3D model allowed for the evaluation of the implants at the site level and the comparison of machined and threaded implant surfaces.

Additional elements such as water irrigation and CG were incorporated to further assess the model. The impact of water irrigation on the results for the US‐PEEK group was investigated through the incorporation of a decontamination group with water‐only. Implants in the water group showed no removal of occlusion spray. Additionally, the mechanism of action for OCB was further investigated by adding the following decontamination groups to the study: OCB with water, OCB with CG, and OCB with BG. Decontamination groups of OCBs with or without water showed similar results with darker green remnant occlusion spray. These findings suggested that the results for the US‐PEEK were not attributed to sonication and not simultaneous water use. The inclusion of chlorhexidine gel was motivated by its frequent clinical use. A BG group was established to determine whether the efficacy in spray removal was due to the gel viscosity or its specific content. When OCB was combined with BG or CG, all OCB groups showed effective removal of the occlusion spray at all four sites compared to the control group (*p* < .05). The fluorescence analysis revealed that OCB combined with CG was more effective in removing occlusion spray than CG alone, both quantitatively and qualitatively. Effective removal of the occlusion spray using the OCB combined with CG can be explained by the isopropanol content in CG and its capacity to dissolve the colored occlusion spray. However, CG alone was inadequate to dissolve a substantial amount of the occlusion spray from the implant surface, as shown when CG was used without OCB. Thus, the combined mechanical action of OCB and the chemical properties of chlorhexidine enhanced the removal of the occlusion spray. This outcome indicates the utility of the 3D model in assessing the efficacy of combined mechanical and chemical decontamination methods.

Efficient decontamination of the threaded implant area was demonstrated for the US‐PEEK group. However, the SEM images showed scratching of the machined implant collar and flattening porosities in the threaded SLA implant area. In contrast, the OCB group showed efficient decontamination of the machined implant neck while preserving intact surface topography. Bacterial colonization on implants is influenced by surface roughness (Louropoulou et al., 2012). The roughness and threaded design make the implant surface more prone to biofilm buildup than natural teeth (Quirynen & van Steenberghe, 1993). The risk is also present on smooth implant surfaces (Quirynen & Bollen, 1995). Scratching of the machined implant area may facilitate biofilm accumulation, potentially leading to inflammation and bone loss in susceptible patients. However, no direct evidence links instrument‐induced roughness to biofilm accumulation (Louropoulou et al., 2012; Monje, Insua, et al., 2019). Furthermore, micromechanical damage to the implant surface may cause corrosion and release of nanoscale particles into surrounding tissues, potentially triggering adverse reactions in peri‐implant tissues (Kotsakis & Olmedo, 2021).

Data from optical profilometry showed no statistically significant differences between the decontamination groups or the implant surfaces. However, the SEM images revealed scratching of the machined implant neck and the threaded area in the US‐PEEK group. The variation between profilometry and SEM results may be attributed to differences in magnification. SEM images were obtained at ×2000, whereas profilometry used a ×20 objective, resulting in larger region of interest containing only few scratches on otherwise intact implant surface that was analyzed by the profilometer. Additionally, the limited number of implants scanned for profilometry is a study limitation.

It is important to acknowledge the limitations of the present study. The absence of model installation in the phantom head before decontamination and the exclusion of supracontructions may impact the applicability of the findings. The incorporation of soft tissue imitation in the model, as suggested by Korello et al. (2023), was not included in the present study. Future research is warranted to incorporate these elements. Nevertheless, the easy removal of implants after decontamination enabled quantitative and qualitative analysis of the implant surface, providing a detailed assessment of the decontamination efficacy.

## 5 CONCLUSION

The present in vitro study describes a promising 3D‐printed model designed for evaluating devices used in the mechanical decontamination of the implant surfaces in advanced peri‐implant defects. The model is anatomically realistic and can easily be modified to mimic different bone defect topographies with high accuracy and reproducibility, providing a standardized method to assess accessibility and implant surface damage. Although no decontamination group achieved complete removal of the colored occlusion spray, the results in the present study showed that the 3D model allowed postdecontamination analysis of implants at the site level. Additionally, changes in the occlusion spray can be used to detect areas with inaccessible and incomplete decontamination. Further studies should explore different peri‐implant defect configurations and incorporate true, dynamic biofilm. Results from in vitro investigations using colored coatings to simulate biofilm should be interpreted cautiously as they present a treatment bias.

## AUTHOR CONTRIBUTIONS

Sadia Nazir Khan, Odd Carsten Koldsland, Hanna Tiainen, and Carl Hjortsjö contributed to the conception, design, data acquisition, and interpretation and drafted and critically revised the manuscript. Sadia Nazir Khan and Hanna Tiainen performed the statistical analyses.

## CONFLICT OF INTEREST STATEMENT

The authors declare no conflict of interest.

## Supporting information

Supporting information.

## Data Availability

The data that support the findings of this study are available from the corresponding author upon reasonable request.
