# Supplementary material for: Anatomical three‐dimensional model with peri‐implant defect for in vitro assessment of dental implant decontamination
Source: Clin Exp Dent Res. 2024 Jan 31;10(1):e841. doi: 10.1002/cre2.841 (PMC10829417; doi:10.1002/cre2.841)
Supplement: Supplementary file 1 — Supporting information. [file CRE2-10-e841-s001.docx]

*Anatomical 3D model with peri-implant defect for in vitro assessment of dental implant decontamination*

**Supporting information**

Sadia N. Khan ^1^, Odd Carsten Koldsland ^2^, Hanna Tiainen ^3^, Carl Hjortsjö ^1^

^1^ Department of Prosthetics and Oral Function, Institute of Clinical Dentistry, Faculty of Dentistry, University of Oslo, Norway

^2^ Department of Periodontology, Institute of Clinical Dentistry, Faculty of Dentistry, University of Oslo, Norway

^3^ Department of Biomaterials, Institute of Clinical Dentistry, Faculty of Dentistry, University of Oslo, Norway

**Correspondence**

Sadia Nazir Khan, DDS, Specialist in Prosthodontics, Department of Prosthetics and Oral Function, Institute for Clinical Dentistry, Faculty of Dentistry, University of Oslo, Norway.

E-mail: [sadiak@odont.uio.no](mailto:sadiak@odont.uio.no)

**Table of Contents**

[Image analysis S3](#_Toc135164373)

[Figure S1 S3](#_Toc135164374)

[Standard curve for fluorescence analysis S4](#_Toc135164375)

[Figure S2 S4](#_Toc135164376)

[Microscopic appearance of occlusion spray layer S5](#_Toc135164377)

[Figure S3 S5](#_Toc135164378)

[Visual appearance of implants following debridement S6](#_Toc135164379)

[Figure S4 S6](#_Toc135164380)

[Figure S5 S7](#_Toc135164381)

# Image analysis


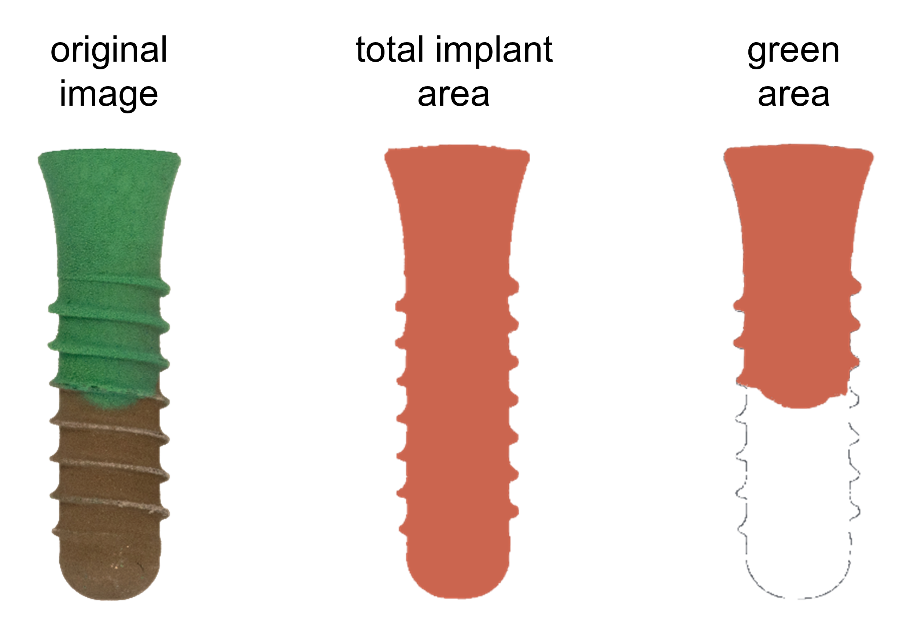


Figure S1: Digital evaluation of the implant area covered with remnant coloured occlusion spray *vs* total implant area using ImageJ®. After adjusting the implant photograph for contrast, the images were binarized based on colour histograms to determine both the total projected implant area and the area covered with the green occlusion spray following decontamination treatment. To compensate for potential minor alterations in implant orientation and imaging angle between experiments and test groups, all quantitative results of the image analysis are presented as ratio between green area and total implant area for each implant in percentage:

$$Green area of total implant area = \frac{green area}{total implant area}\times100\%$$

# Standard curve for fluorescence analysis


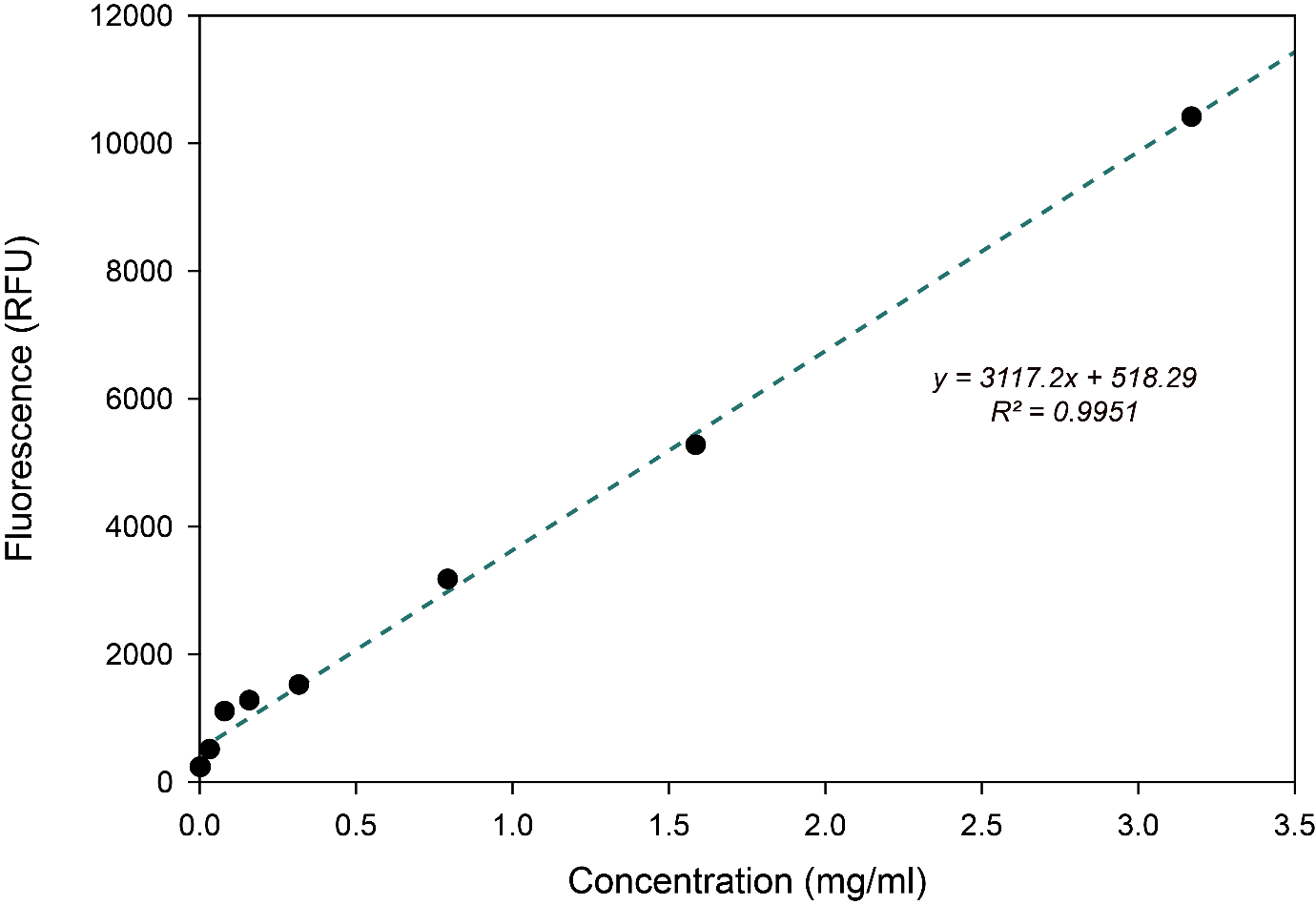


Figure S2: A standard curve was generated by recording the relative fluorescence at far red (665-720 nm) emission wavelength of serial dilutions of known concentrations of the used occlusion spray dissolved in isopropanol using blue LED excitation at 470 nm to confirm linear relationship between measured fluorescence and the concentration of occlusion spray. Autofluorescence from chitosan was not observed at the used excitation/emission wavelengths.

# Microscopic appearance of occlusion spray layer


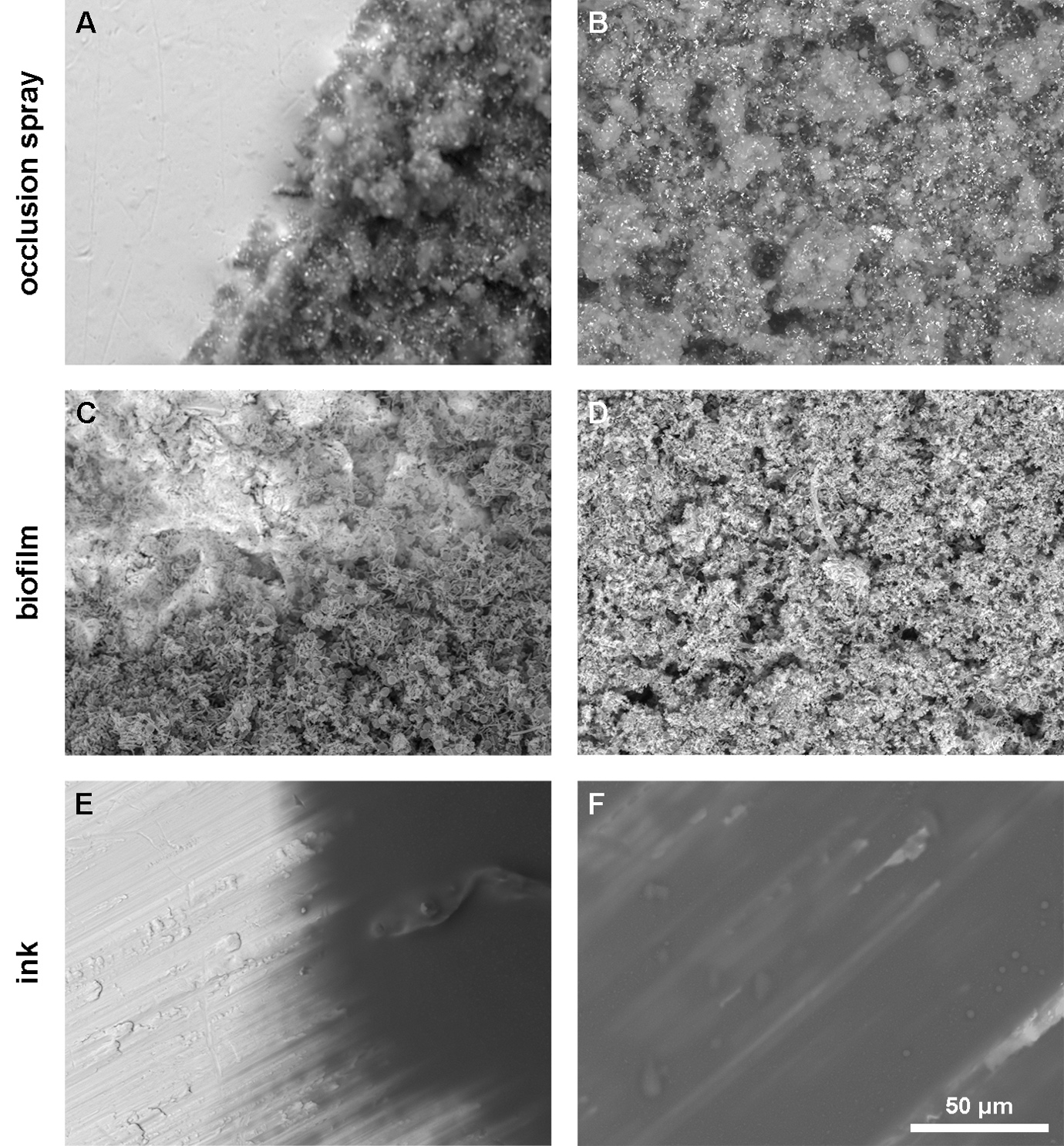


Figure S3: A) Thickness of the occlusion spray layer was determined by measuring the average step height between the occlusion spray layer and implant surface that had been partially masked while the occlusion spray was applied using optical profilometry. B) The occlusion spray consisted of inorganic microparticles embedded within a polymeric matrix and morphologically resembled multispecies biofilm grown from saliva on titanium implant surface for 72 hours in anaerobic conditions (C and D). Occlusion spray layer also demonstrated 3D characteristics that were similar to biofilm, compared to the 2D appearance of an ink layer (Staedtler Lumocolor permanent black) applied on titanium surfaces by dipping the titanium samples in the ink (E and F).

# Visual appearance of implants following debridement

***Mechanical decontamination***


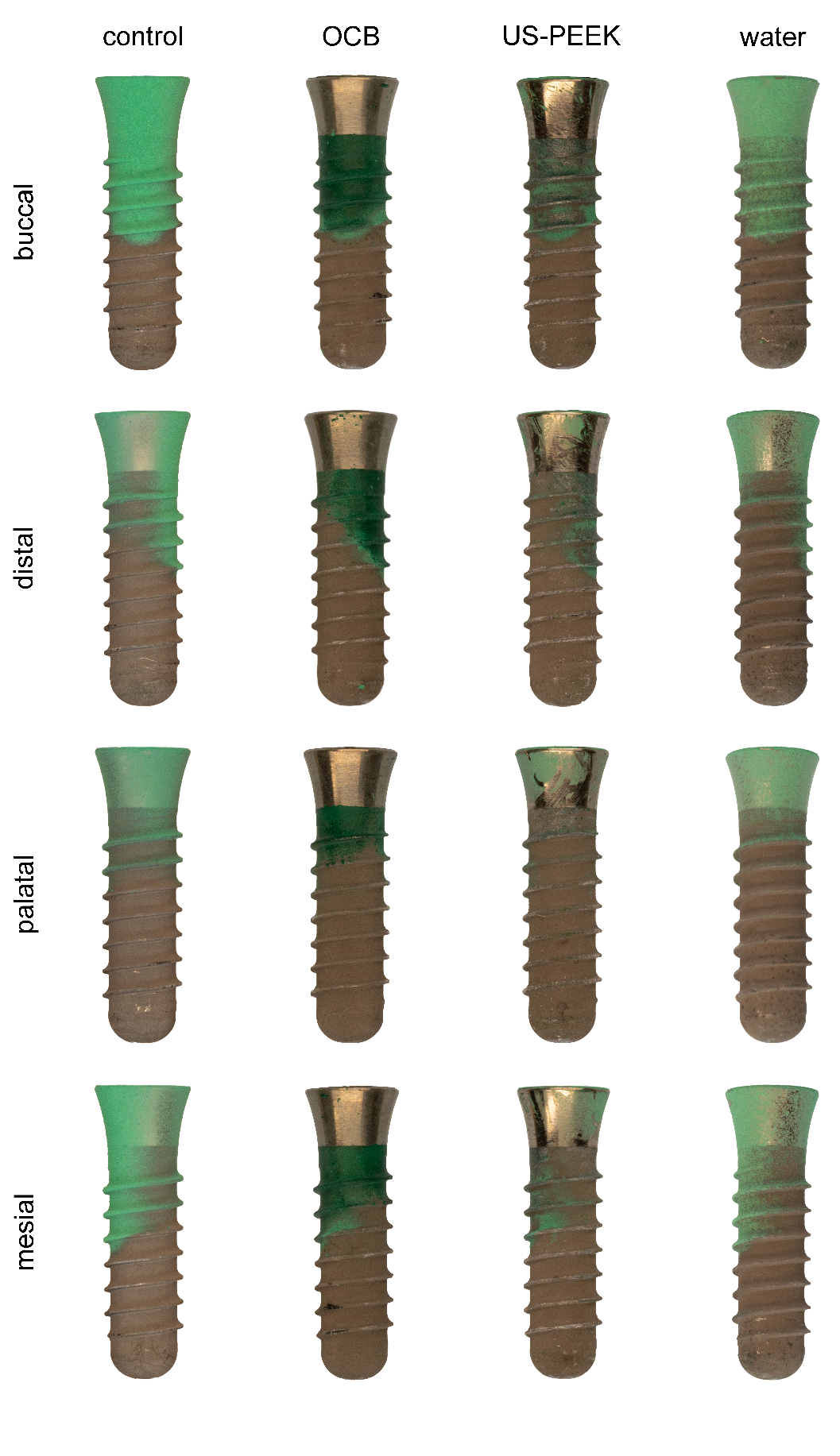


Figure S4: Images of the control and the mechanically decontaminated implants obtained from the buccal, distal, palatal, and mesial surfaces. The apical termination of the green occlusion spray shows that the spray did not spread to the unexposed implant surface. The apical spray pattern also indicates that the defect was identical in all models.

***Mechanical and chemical decontamination***


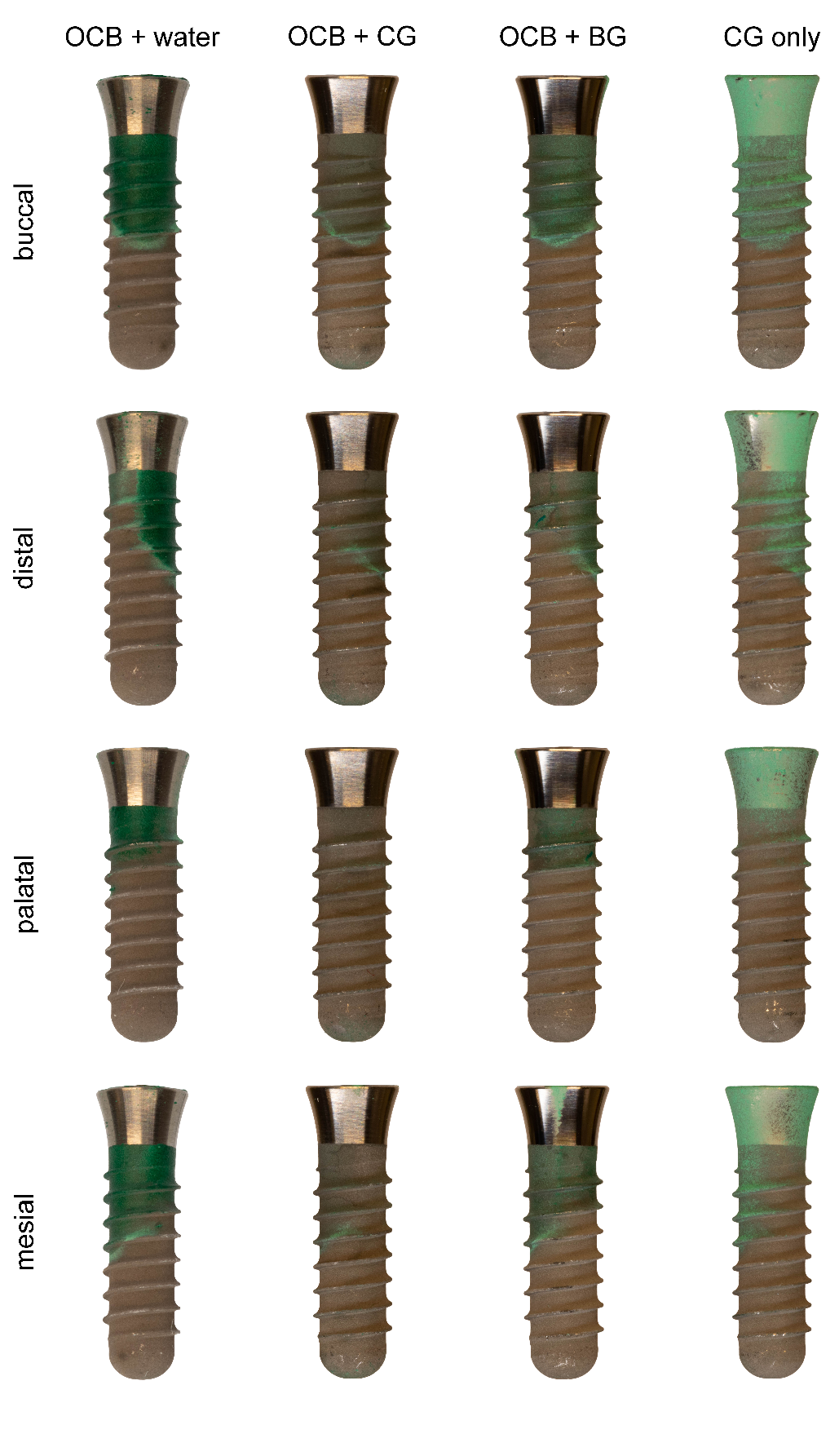


Figure S5: Images obtained from the buccal, distal, palatal, and mesial showing changes in the coloured occlusion spray after chemical or combined mechanical and chemical decontamination treatment.
